# Thirty-year changes in stroke demographics in the Saudi National Guard Community (1982–1992 and 2013–2024)

**DOI:** 10.3389/fneur.2026.1806977

**Published:** 2026-05-26

**Authors:** Awadh M. Alahmari, Alwaleed Alabdulwahed, Abdulrahman A. Almasood, Eman Alotaibi, Maha Albakr, Lena Alotaibi, Ismail A. Khatri

**Affiliations:** 1 Department of Neurology, King Abdulaziz Medical City, MNGHA, Riyadh, Saudi Arabia; 2 King Saud Bin Abdulaziz University for Health Sciences (KSAU-HS), Riyadh, Saudi Arabia; 3 King Abdullah International Medical Research Center (KAIMRC), MNGHA, Riyadh, Saudi Arabia

**Keywords:** stroke epidemiology, Saudi Arabia, incidence and prevalence, TOAST classification, vascular risk factors, stroke outcomes, National Guard Community, ischemic stroke

## Abstract

**Background:**

Stroke epidemiology in Saudi Arabia has evolved substantially over three decades due to advances in clinical care, diagnostic technology, and demographic change. This study descriptively compares stroke characteristics, risk factors, and outcomes in the Saudi National Guard (SANG) community across two historical periods, acknowledging important methodological constraints on cross-period comparability.

**Methods:**

A retrospective comparative study analyzed 3,436 stroke patients from June 2013 to January 2024 at King Abdulaziz Medical City, Riyadh (primary registry data), compared with 500 patients from December 1982 to June 1992 [secondary data derived from Al Rajeh et al.]. Patients with transient ischemic attack or stroke mimickers were excluded. Variables included demographics, vascular risk factors, stroke type (TOAST classification with retrospective mapping for the historical period), crude incidence, prevalence, mortality, and — for the Modern Era only — functional outcome (mRS 0–2 at 3 months) and length of stay (LOS). A new age-stratified analysis was performed using individual-level data from 3,434 Modern Era patients. All risk ratios (RRs) are unadjusted descriptive estimates.

**Findings:**

Crude annual stroke incidence appears to have decreased from 43.8 to 34.36 per 100,000 (unadjusted RR 0.79, 95% CI 0.67–0.92), and 10-year period prevalence increased from 186 to 343.6 per 100,000. Importantly, the Modern Era cohort had a lower mean age (59.8 ± 13.3 years) than the historical cohort (63 years), indicating the apparent incidence decline is not attributable to demographic aging. Risk factors increased substantially: hypertension (72.1% vs. 56.0%), diabetes mellitus (64.1% vs. 42.0%), and smoking (16.9% vs. 6.0%). Intracerebral hemorrhage declined (14.6% vs. 21.4%). All-cause mortality fell from 12.2 to 4.2% (unadjusted RR 0.34, 95% CI 0.25–0.46), the sole outcome for which direct cross-period comparison is feasible. In the Modern Era, 56.8% of patients with available data achieved mRS 0–2 at 3 months (descriptive, no historical comparator).

**Interpretation:**

These descriptive observations are consistent with global patterns of declining stroke mortality and rising metabolic risk burden. All cross-period comparisons must be interpreted cautiously given differences in diagnostic technology, data sources, population composition, and the absence of age-sex standardized rates. The rising metabolic risk factor burden underscores the urgent need for targeted primary prevention strategies in this community.

## Introduction

Stroke, characterized by interrupted blood flow to the brain due to ischemia or hemorrhage, is a leading cause of global mortality and disability, with over 12.2 million new cases and 110 million survivors annually (1). It imposes significant social and economic burdens (2). Globally, stroke is the second leading cause of death and disability-adjusted life years, with approximately 5.8 million stroke-related deaths annually (3, 4).

In Saudi Arabia, a 1993 community-based survey reported a crude stroke incidence of 43.8 per 100,000, attributed partly to a younger population structure at the time (5). A 2002 review noted a stroke-type distribution broadly similar to Western countries but with lower crude incidence and prevalence (6). More recent data from the Aseer region report higher incidence (57.64 per 100,000) with projections of continued rise (7, 8).

The Saudi Arabian National Guard (SANG) community — comprising soldiers and their dependents — receives healthcare through a dedicated network of facilities, of which King Abdulaziz Medical City, Riyadh is the largest. Over 30 years, this community expanded from approximately 120,000 to approximately 1,000,000 members, and has experienced substantial changes in demographic profile, vascular risk factors, and access to advanced stroke care including dedicated stroke units and thrombolytic therapy.

This study presents a descriptive comparison of stroke epidemiology across two periods — December 1982 to June 1992 and June 2013 to January 2024 — using available historical published data and prospective registry data from this defined population. Given the inherent methodological constraints of cross-era comparison (detailed in the Limitations section), findings are presented as descriptive observations rather than definitive epidemiological trends.

## Methods

### Study design

Retrospective comparative descriptive study. The Modern Era cohort (2013–2024, *n* = 3,436) comprises primary prospective hospital registry data collected at King Abdulaziz Medical City. The Historical Era data (1982–1992, *n* = 500) are derived from a previously published study of the same institutional population (5), representing secondary aggregate data. This data-source asymmetry is a recognized limitation addressed in the Limitations section.

### Population

All patients with confirmed acute stroke admitted to King Abdulaziz Medical City were included. Patients with transient ischemic attack or stroke mimickers were excluded. The study population is drawn from the SANG community, a defined and largely closed healthcare system.

### Data collection

Modern Era data were extracted from the institutional stroke service database and discharge records using a standardized form. Variables included age, sex, nationality, vascular risk factors (hypertension: BP ≥ 140/90 mmHg or antihypertensive medication; diabetes mellitus: fasting glucose >6 mmol/L or HbA1c > 6.5%; smoking [current and ex-smoker]; cardiac conditions including arrhythmia and valvular heart disease), stroke type, crude incidence, prevalence, mortality, mRS 0–2 at 3 months, and LOS (stays >30 days for social or chronic care reasons excluded).

### Stroke classification

Modern Era strokes were classified using the TOAST system (large vessel atherosclerosis, small vessel occlusion, cardioembolic, undetermined, other determined etiology; intracerebral hemorrhage; cerebral venous thrombosis). Historical Era cases were originally classified using a pre-TOAST system from Al Rajeh et al. (5) and were mapped to TOAST-equivalent categories for this comparison. This mapping introduces uncertainty, particularly for ischemic subtypes; all subtype comparisons should be considered exploratory.

### Age-stratified analysis

Individual-level age data were available for 3,434 of 3,436 Modern Era patients. Patients were grouped into six age bands (<40, 40–49, 50–59, 60–69, 70–79, ≥80 years) and descriptively characterized to contextualize the crude incidence comparison against the historical cohort mean age of 63 years.

### Statistical analysis

Categorical variables were compared using Chi-square or z-tests (significance threshold *p* < 0.05). Unadjusted RRs with 95% confidence intervals (CIs) were calculated using Poisson regression (for incidence comparisons) and Chi-square tests (for categorical outcomes). All reported RRs are unadjusted for age, sex, or comorbidities and represent crude between-period associations. Multivariate adjustment across periods was not feasible due to the aggregate-only nature of the historical data.

## Results

### Demographics

The Historical Era cohort (n = 500) comprised 68.4% males and 31.6% females; all were Saudi nationals. The Modern Era cohort (n = 3,436) comprised 66.5% males (n = 2,286) and 33.3% females (n = 1,144), with 6 patients missing gender data; 12.1% were non-Saudi residents (n = 413 of 3,412 with complete nationality data). Sex distribution was statistically similar between eras (*p* = 0.4377). In the Modern Era, females were significantly older at stroke onset than males (mean 61.3 ± 14 vs. 55.1 ± 14 years; *p* < 0.0001). (Table 1).

**Table 1:** Demographics.

Characteristic	1982–1992 (*n* = 500)	2013–2024 (*n* = 3,436)	*p*-value
Age, mean (SD), years	63 (17)	59.8 (13.3)*	<0.0001†
Male, No. (%)	342 (68.4)	2,286 (66.5)	0.4377
Female, No. (%)	158 (31.6)	1,144 (33.3)‡	0.5143
Saudi Nationality, No. (%)	500 (100)	2,999 (87.9)§	—
Non-Saudi Nationality, No. (%)	0 (0)	413 (12.1)§	—

*Modern Era mean age from individual-level data (*n* = 3,434; 2 patients missing age). ^†^*p*-value for Modern Era age reflects male vs. female difference (55.1 ± 14 vs. 61.3 ± 14 years). ^‡^Six patients with missing gender data; gender-complete records total 3,430. ^§^24 patients with missing nationality data excluded from nationality analysis.

### Age distribution of the modern era cohort

Individual-level age data were available for 3,434 Modern Era patients (mean 59.8 ± 13.3 years, median 61 years). The complete age-group distribution is presented in Table 2. The largest group was aged 60–69 years (n = 992, 28.9%), followed by 50–59 years (n = 829, 24.1%) and 70–79 years (n = 756, 22.0%). Patients under 40 years constituted 7.5% of the cohort. Overall, 60.7% of Modern Era stroke patients were under 65 years of age. The Modern Era mean age (59.8 years) is lower than the historical cohort mean of 63 years, a finding with important implications for interpreting the crude incidence comparison (see Discussion).

**Table 2:** Age distribution of modern era stroke cohort (2013–2024)—new analysis.

Age group	*N*	% of Cohort	Contextual note
< 40 years	259	7.5%	Stroke at early age; includes young adults
40–49 years	467	13.6%	Middle-aged
50–59 years	829	24.1%	Second largest group
60–69 years	992	28.9%	Largest group in this cohort
70–79 years	756	22.0%	Older adults
≥80 years	131	3.8%	Oldest old
TOTAL	3,434	100%	Mean 59.8 ± 13.3 years; Median 61 years

*n* = 3,434 (2 patients missing age data excluded). Historical Era overall mean age: 63 years ((5)). The Modern Era cohort is on average younger than the historical cohort (mean 59.8 vs. 63 years), with 60.7% of patients under 65 years of age. This age-structure difference indicates the observed crude incidence decrease is not attributable to a younger presenting population and is directionally consistent with genuine improvements in primary stroke prevention, although formal age-sex standardized rates could not be calculated.

### Crude incidence and prevalence

Crude annual stroke incidence appears to have decreased from 43.8 per 100,000 (1982–1992) to 34.36 per 100,000 (2013–2024) (unadjusted RR 0.79, 95% CI 0.67–0.92, *p* < 0.0001). This comparison is constrained by differences in population size, age structure, nationality composition, ascertainment method, and diagnostic technology; it should be interpreted as a crude descriptive estimate only (see Limitations). The 10-year period prevalence increased from 186 to 343.6 per 100,000 (unadjusted RR 1.85, 95% CI 1.56–2.19, *p* < 0.0001), consistent with improved survival in a community that expanded eightfold. (Table 3).

**Table 3:** Crude incidence and prevalence (unadjusted descriptive estimates).

Metric	1982–1992	2013–2024	Unadj. RR (95% CI)	*p*-value
Crude annual incidence (per 100,000)	43.8	34.36	0.79 (0.67–0.92)	<0.0001
10-year period prevalence (per 100,000)	186	343.6	1.85 (1.56–2.19)	<0.0001

All rates are crude and unadjusted for age or sex. Interpretation is limited by differences in population size, age structure, nationality composition, ascertainment method, and diagnostic technology. See Table 2 for age-stratified context.

### Risk factors

All major vascular risk factors increased between eras (all unadjusted comparisons). Hypertension: 72.1% vs. 56.0% (unadjusted RR 1.29, 95% CI 1.14–1.46, *p* < 0.0001); diabetes mellitus: 64.1% vs. 42.0% (RR 1.53, 95% CI 1.32–1.76, p < 0.0001); smoking (current + ex-smoker combined): 16.9% vs. 6.0% (RR 2.81, 95% CI 1.95–4.05, p < 0.0001). Cardiac conditions remained stable (13.0% vs. 13.0%; RR 1.00, *p* = 1.000). These differences may also partly reflect improved ascertainment and more systematic screening in the modern period. (Table 4).

**Table 4:** Stroke types and risk factors (unadjusted comparisons).

Characteristic	1982–1992 (*n* = 500)	2013–2024 (*n* = 3,436)	Unadj. RR (95% CI)	*P*-value
Stroke types				
Ischemic stroke, No. (%)	381 (76.2)	2,800 (81.5)	1.07 (1.01–1.13)	0.0205
Large vessel stroke^†^, No. (%)	31 (6.2)*	969 (28.2)	3.41 (2.38–4.89)†	<0.0001
Small vessel occlusion, No. (%)	121 (24.2)	974 (36.2)	—	0.052
Cardioembolic, no. (%)	260 (52.0)	447 (16.6)	—	<0.001
Other/undetermined, No. (%)	Not reported	294 (10.9)	—	—
Intracerebral hemorrhage, No. (%)	107 (21.4)	502 (14.6)	0.68 (0.56–0.84)	<0.001
Cerebral venous thrombosis, No. (%)	Not reported	131 (3.8)	—	—
Risk factors				
Hypertension, No. (%)	280 (56.0)	2,478 (72.1)	1.29 (1.14–1.46)	<0.0001
Diabetes mellitus, No. (%)	210 (42.0)	2,201 (64.1)	1.53 (1.32–1.76)	<0.0001
Smoking (current + ex-smoker), No. (%)	30 (6.0)	579 (16.9)	2.81 (1.95–4.05)	<0.0001
Cardiac conditions, No. (%)	65 (13.0)	448 (13.0)	1.00 (0.78–1.29)	1.0000

All RRs are unadjusted crude estimates. *Historical LVS proportion estimated indirectly from published aggregate data. †LVS comparison is exploratory: historical data were estimated indirectly, TOAST was applied retrospectively, and modern vascular imaging (CTA/MRA) markedly improves LVS detection vs. CT-only assessment. LVS = large vessel stroke (large-artery atherosclerosis per TOAST).

### Stroke types

Ischemic strokes were predominant in both eras (81.5% vs. 76.2%; unadjusted RR 1.07, 95% CI 1.01–1.13, *p* = 0.0205). The large vessel stroke (LVS) proportion appeared markedly higher in the Modern Era (28.2% vs. 6.2%); this comparison is presented as an exploratory observation only, given indirect historical estimation, retrospective TOAST mapping, and the substantially superior vascular imaging capabilities of the modern period (CT angiography, MR angiography). Intracerebral hemorrhage declined (14.6% vs. 21.4%; unadjusted RR 0.68, 95% CI 0.56–0.84, *p* < 0.001), consistent with improved blood pressure control. Cerebral venous thrombosis constituted 3.8% of Modern Era cases (n = 131) and was not reported in the historical dataset. (Table 4).

### Mortality and modern era outcomes

All-cause in-hospital mortality decreased from 12.2 to 4.2% (unadjusted RR 0.34, 95% CI 0.25–0.46, *p* < 0.0001). This is the sole outcome for which direct cross-period comparison is feasible. The mortality reduction likely reflects improved acute stroke management, though enhanced detection of milder strokes in the modern period may have contributed via a denominator effect.

Functional outcome (mRS 0–2 at 3 months) and LOS are reported for the Modern Era only, as no comparable historical data exist. Among 3,121 patients with available mRS data, 56.8% (n = 1,772) achieved mRS 0–2. Mean LOS was 5.73 days (ischemic stroke), 6.59 days (ICH), and 8.14 days (CVT). These figures are presented as descriptive benchmarks and should not be interpreted as evidence of temporal improvement in functional outcomes. (Table 5).

**Table 5:** Modern era outcomes by stroke type (2013–2024 only—no historical comparator available for mRS or LOS).

Stroke type	N	Mean LOS (days)	mRS 0–2, No. (%)	In-hospital mortality, No. (%)
Ischemic stroke	2,800	5.73	1,713 (61.2)	118 (4.2)*
Large vessel stroke	969	5.73	593 (61.2)	41 (4.2)
Intracerebral hemorrhage	502	6.59	219 (43.6)	20 (4.0)
Cerebral venous thrombosis	131	8.14	67 (51.1)	5 (3.8)

mRS 0–2 at 3 months = good functional outcome. Overall mRS 0–2: 56.8% (n = 1,772/3,121 patients with available mRS data; 315 missing). LOS excludes stays >30 days. *Overall ischemic stroke mortality compared cross-period to historical all-cause mortality (12.2%): unadjusted RR 0.34 (95% CI 0.25–0.46, *p* < 0.0001) — the sole feasible cross-period outcome comparison. mRS and LOS are descriptive Modern Era benchmarks only; no historical data are available for comparison.

## Discussion

This study presents descriptive observations on stroke epidemiology in the SANG community across two periods approximately 30 years apart. The findings are broadly consistent with global patterns — declining crude mortality, rising metabolic risk factors, and increasing stroke prevalence driven by population growth and improved survival — but must be interpreted within the methodological constraints inherent to historical comparison.

The apparent decrease in crude annual incidence (43.8 to 34.36 per 100,000) is consistent with global trends attributable to improved risk factor management (9). A new age-stratified analysis using individual-level data from 3,434 Modern Era patients reveals that the modern stroke population has a lower mean age (59.8 years) than the historical cohort (63 years), with 60.7% of patients under 65 years. Since younger populations are expected to exhibit lower stroke incidence even in the absence of prevention gains, the observed crude incidence decrease despite a younger presenting population is directionally consistent with genuine improvements in primary prevention rather than being explicable by demographic aging alone. We acknowledge that formal age-sex standardized incidence rates cannot be calculated and that this argument is contextual rather than conclusive.

The observed rise in ischemic stroke proportion (76.2 to 81.5%) and the escalating prevalence of hypertension, diabetes, and smoking are consistent with documented lifestyle transitions toward Westernized dietary patterns and sedentary behavior in the MENA region (10). The increasing metabolic risk factor burden is a major public health concern independent of the limitations affecting precise trend quantification. The apparent large increase in large vessel stroke proportion (6.2 to 28.2%) is most likely substantially attributable to improved vascular imaging and retrospective TOAST mapping, and is presented as an exploratory observation only.

The mortality reduction (12.2 to 4.2%) is the most robust inter-period finding, as it relies on a relatively consistent outcome definition. This decline likely reflects advances in acute stroke management, including dedicated stroke units and thrombolytic therapy, in alignment with Saudi Vision 2030 health infrastructure goals (11, 12). The concurrent detection of milder strokes via advanced neuroimaging may have contributed a denominator effect. Gender-specific patterns — notably older age at stroke onset in females (61.3 vs. 55.1 years, *p* < 0.0001) — are consistent with patterns reported across Middle Eastern populations (13) and warrant targeted gender-sensitive prevention strategies.

National stroke registries with prospective, standardized individual-level data collection are essential for accurately monitoring epidemiological trends and evaluating prevention programs in Saudi Arabia (14). The rising burden of large vessel stroke necessitates expanded access to mechanical thrombectomy and advanced stroke unit care.

## Limitations

### Heterogeneous data sources

Historical data are secondary aggregate data (5); Modern Era data are primary registry records. Potential inconsistencies in variable definitions, diagnostic criteria, and data quality limit direct comparability.

### Crude rates only—no age-sex standardization

Age-sex standardized incidence rates could not be calculated due to unavailability of historical denominator data by age and sex. All rate comparisons are crude descriptive estimates. The new age-distribution analysis (Table 2) contextualizes the demographic structure of the Modern Era cohort but does not substitute for formal standardization.

### Diagnostic and detection bias

The widespread adoption of MRI with diffusion-weighted imaging in the Modern Era enables detection of smaller ischemic lesions than CT-predominant diagnostics in the 1980s–1990s. This technological shift likely inflated the apparent ischemic proportion and may have contributed to lower observed mortality via a milder-case denominator effect (see Table 6).

**Table 6:** Comparability framework: systematic characterisation of differences between the two study periods.

Dimension	1982–1992	2013–2024	Impact on comparability
Sample size	500	3,436	Larger modern sample; not directly comparable
Population denominator	~120,000	~1,000,000	Eightfold expansion; crude rates computed against separate denominators
Nationality composition	100% Saudi	87.9% Saudi, 12.1% non-Saudi	Non-Saudi inclusion may affect risk profile; discussed in Limitations
Data source	Secondary (Al Rajeh et al.)	Primary prospective registry	Asymmetry in data quality and variable definitions
Diagnostic technology	CT predominant	MRI/DWI, CTA, MRA	Detection bias for small ischemic lesions and LVS; directly affects subtype proportions
Prevalence definition	Community-based (186/100,000)	10-year period (343.6/100,000)	Different methodologies; comparison is descriptive only
Age structure	Mean 63 yrs. (aggregate)	Mean 59.8 ± 13.3 yrs. (individual)	Modern cohort is younger; contextualises crude incidence comparison
Stroke classification	Pre-TOAST (retrospectively mapped)	Prospective TOAST	Subtype comparisons exploratory only; LVS estimated indirectly for historical era
Outcome data availability	Mortality only	Mortality, mRS, LOS	Cross-period comparison feasible for mortality only; mRS and LOS are Modern Era descriptive benchmarks

LVS, large vessel stroke; MRI, magnetic resonance imaging; DWI, diffusion-weighted imaging; CTA, CT angiography; MRA, MR angiography; mRS, modified Rankin Scale; LOS, length of stay. This framework is provided to ensure transparency about the limitations of cross-era comparison. All between-period comparisons in this manuscript are descriptive estimates subject to the constraints listed above.

### TOAST classification mapping

TOAST was published in 1993. Historical stroke subtypes were mapped retrospectively from the classification used by Al Rajeh et al. (5). The large vessel stroke proportion in the historical era was derived indirectly. All stroke subtype comparisons are therefore exploratory.

### Hospital-based ascertainment

Both cohorts are hospital-based. Cases with fatal prehospital events or those managed at other facilities may not be captured. Changes in emergency medical services and health-seeking behavior over 30 years may differentially affect ascertainment between periods.

### Unadjusted analyses

All between-period RRs are unadjusted for age, sex, or comorbidities. Residual confounding is likely substantial. Multivariate adjustment was not feasible given the aggregate nature of historical data.

### Non-Saudi population in the modern era

Inclusion of 12.1% non-Saudi residents (absent in the historical cohort) introduces a comparability limitation, as this group may differ in age structure, risk factor prevalence, and care-seeking patterns.

### Missing outcome data for the historical period

mRS and LOS data are available for the Modern Era only. In-hospital mortality is the sole cross-period outcome comparison that is feasible.

## Conclusion

This descriptive comparative study suggests that all-cause stroke mortality has declined substantially over 30 years in the SANG community, while metabolic risk factors have increased markedly and stroke prevalence has risen, consistent with improved survival in a rapidly growing population. Crude incidence appears lower in the modern period; a new age-stratified analysis demonstrates that the Modern Era stroke population is younger on average than the historical cohort, indicating this decrease is not explained by demographic aging. All cross-period comparisons should be interpreted as descriptive observations given the inherent methodological constraints of historical comparison. These findings highlight the critical need for targeted primary prevention strategies addressing hypertension, diabetes, and smoking; continued investment in advanced stroke care infrastructure aligned with Saudi Vision 2030; and establishment of national prospective stroke registries to enable accurate epidemiological monitoring.

## Data Availability

The original contributions presented in the study are included in the article/supplementary material, further inquiries can be directed to the corresponding author.
